# The Association between Melatonin-Containing Foods Consumption and Students’ Sleep–Wake Rhythm, Psychoemotional, and Anthropometric Characteristics: A Semi-Quantitative Analysis and Hypothetical Application

**DOI:** 10.3390/nu15153302

**Published:** 2023-07-25

**Authors:** Mikhail F. Borisenkov, Sergey V. Popov, Vasily V. Smirnov, Ekaterina A. Martinson, Svetlana V. Solovieva, Lina A. Danilova, Denis G. Gubin

**Affiliations:** 1 Department of Molecular Immunology and Biotechnology, Institute of Physiology of Komi Science Centre of the Ural Branch of the Russian Academy of Sciences, Syktyvkar 167982, Russia; s.v.popov@inbox.ru (S.V.P.); smirnowich@yandex.ru (V.V.S.); 2 Institute of Biology and Biotechnology, Vyatka State University, Kirov 610000, Russia; biotech.vgu@gmail.com; 3 Department of Biology, Tyumen Medical University, Tyumen 625023, Russia; svsolov@mail.ru (S.V.S.); danic_72@mail.ru (L.A.D.); 4 Laboratory for Chronobiology and Chronomedicine, Research Institute of Biomedicine and Biomedical Technologies, Tyumen Medical University, Tyumen 625023, Russia; 5 Tyumen Cardiology Research Centre, Tomsk National Research Medical Center, Russian Academy of Science, Tyumen 119991, Russia

**Keywords:** food melatonin, chronotype, social jetlag, sleep quality, depression, schoolchildren, university students

## Abstract

Food is an important source of melatonin (MT), which belongs to a group known as chronobiotics, a class of substances that affect the circadian system. Currently, no studies have been conducted on how the consumption of foods containing MT (FMT) is associated with indicators characterizing the human circadian system. In this study, we tested the hypothesis that FMT consumption is associated with chronotype and social jetlag. A total of 1277 schoolchildren and university students aged *M* (SD) 19.9 (4.1) years (range: 16–25 years; girls: 72.8%) participated in a cross-sectional study. Each participant completed an online questionnaire with their personal data (sex, age, height, weight, waist circumference, and academic performance) and a sequence of tests to assess their sleep–wake rhythm (the Munich Chronotype Questionnaire), sleep quality (the Pittsburgh Sleep Quality Index), and depression level (the Zung Self-Rating Depression Scale). Study participants also completed a modified food frequency questionnaire that only included foods containing MT (FMT). They were asked how many foods containing MT (FMT) they had eaten for dinner, constituting their daily serving, in the past month. The consumption of foods containing MT (FMT) during the day (FMT_day_) and at dinner (FMT_dinner_) was assessed using this test. Multiple regression analyses were performed to assess the association between the studied indicators. We found that higher FMT_day_ values were associated with early chronotype (*β* = −0.09) and less social jetlag (*β* = −0.07), better sleep quality (*β* = −0.06) and lower levels of depression (*β* = −0.11), as well as central adiposity (*β* = −0.08). Higher FMT_dinner_ values were associated with a lower risk of central adiposity (*β* = −0.08). In conclusion, the data obtained confirm the hypothesis that the consumption of foods containing MT (FMT) is associated with chronotype and social jetlag in adolescents and young adults.

## 1. Introduction

The circadian system (CS) in mammals, formed during evolution, is present in all species of organisms living on the Earth’s surface [1], including humans [2]. Its main function is maintaining the circadian rhythms of biochemical, physiological, and psychological processes and synchronizing endogenous rhythms with 24-h environmental rhythms [3]. Retinal ganglion cells containing the pigment melanopsin perceive the daily rhythm of illumination, which is the primary external synchronizing signal for the CS [4]. From there, along the retinohypothalamic tract, the signal is transmitted to the suprachiasmatic nuclei (SCN) of the hypothalamus. In the SCN, the autonomous circadian rhythm of electrical activity synchronizes with the 24-h rhythm of illumination. Furthermore, information on the daylight period is transmitted using neurohumoral signals to the underlying peripheral organs. Signal transduction from the SCN to peripheral organs is mediated by the hormone melatonin (MT), which is synthesized by the pineal gland in antiphase with daylight rhythm, thus defining the boundaries of “biological night” [5].

The important role of CS in human life has been repeatedly confirmed [2]. Over the past few hundred years, the human environment has dramatically changed. Most humans live in cities and spend most of their time indoors, and artificial lighting has become widespread. As a result, the role of climatic factors in human life has significantly decreased, and the role of social factors has increased. This shift has affected the function of human CS. Recently, a specific impairment of human CS function called social jetlag (SJL) [6] has been described. SJL, which is more common in late chronotype individuals, is caused by an imbalance between sleep–wake and social life rhythms [7]. SJL is most often detected between the ages of 16 and 18 [8] when puberty-induced sleep–wake phase delay reaches its maximum and leads to adolescents’ inability to adapt to school schedules [9]. SJL has been associated with decreased academic performance [10], cognitive function [11], increased depression risk [12], and obesity [13].

Thus, the human CS cannot often adapt to life in a social environment, which leads to negative consequences. Therefore, it is necessary to use additional mechanisms to maintain its functional state, including nutrition and food regulation. Food has been actively studied as a source of chronobiotics, a class of substances that regulate CS function [14]. One of the best-known chronobiotics is MT [5]. Knowledge has increased regarding the content of MT in foods of plant and animal origin [15]. Significant levels of FMT have been noted in some food products, such as cherries and walnuts [16,17]. In experimental studies, FMT consumption has been associated with an increase in blood MT levels [17,18], improved sleep function [19,20,21,22,23], and psychoemotional state [24]. However, in some studies [25,26], FMT consumption had no positive effect on sleep function. The reason for this may be that these studies investigated the effect of adding certain foods with known FMT content to the diet. However, the total dietary intake of FMT was not controlled. To date, only one study has examined the effect of total FMT intake on the human organism [27]. The authors found an inverse association between total FMT consumption and all-cause mortality risk. In the available literature, there is no information on FMT’s effect on human CS.

The purpose of this study was to test whether FMT-containing foods consumption is associated with indicators characterizing the human circadian system. To achieve this goal, the following tasks were set: (a) to estimate the total FMT content in food consumed by a person during the day and at dinner; (b) to study associations between FMT consumption and the sleep–wake rhythm (chronotype, social jetlag, sleep duration, sleep quality) as well as cognitive (academic performance), psychoemotional (depression), and anthropometric (body mass index, central adiposity) characteristics.

## 2. Materials and Methods

### 2.1. Objectives and Study Design

The online survey was conducted from November 2021 to December 2022 and involved the anonymous and voluntary participation of secondary school students from the Komi Republic and university students from Syktyvkar, Kirov, and Tyumen, Russia. Teachers and school psychologists distributed information regarding the study. Exclusion criteria included clinically diagnosed sleep disorders, eating disorders, and night shift work. Eligible participants included male and female schoolchildren and university students aged 16–25 who provided their informed consent. Out of 1455 invitations distributed, 178 people (12%) refused to participate in the survey or did not complete most of the questionnaires. The final database included 1277 questionnaires.

This study was approved by the Ethics Committee of the Institute of Physiology of the Komi Science Centre of the Ural Branch of the Russian Academy of Sciences (Protocol #6, 21.09.2020). Verbal informed consent was obtained from all study participants. Additionally, schoolchildren’s parents provided written informed consent.

### 2.2. Instruments

Each study participant provided personal information and completed the Munich Chronotype Questionnaire (MCTQ) [7], Zung Self-Rating Depression Scale (ZSDS) [28], and Pittsburgh Sleep Quality Index (PSQI) [29].

#### 2.2.1. Personal Data

The study participants were asked to specify their place of residence, sex, age, academic performance, height, weight, and waist circumference. Weight and height were used to evaluate body mass index (BMI), calculated as weight in kilograms divided by height in meters squared. Sex- and age-adjusted BMI percentiles (BMI%) were determined using BMI growth charts [30]. Four BMI categories (BMIc) were defined according to World Health Organization criteria [30]: (1) underweight (*n* = 101); (2) normal weight (*n* = 1003); (3) overweight (*n* = 122); and (4) obese (*n* = 51). The waist circumference to height ratio (WHtR) was also calculated [31]. Since the study began near the end of the third wave of the COVID-19 pandemic, some participants (*n* = 349) completed the questionnaires while remote learning. We adjusted our data analysis to reflect the significant alterations to sleep–wake patterns caused by remote learning [32].

#### 2.2.2. Academic Performance

To assess academic performance, all participants were asked the following question: ‘What was your academic performance (GPA) for the quarter or session preceding the study?’. In Russia, a unified, coded grading system for schoolchildren and university students is used. It consists of five grade points. Scores “1” and “2” correspond to unsatisfactory (requiring retakes of exams), “3”—low, “4”—average, and “5”—high academic performance. The mean GPA value among the study participants was *M* (SD): 4.30 (0.51).

#### 2.2.3. MCTQ

The test questions concerned sleep onset time, awakening time on weekdays and free days, the use of an alarm clock, and the length of the school week. Based on these data, the following indicators were calculated: chronotype (MSF_SC_), social jetlag (SJL), average weekly sleep duration (SlD), and sleep efficiency (SlE). The formulas and calculation methods for the indicators listed above were also described in Borisenkov et al. [33]:

SlD = (SlDw × [7 − FD] + SlD_F_ × FD)/7 (1)

SlE = TiB/SlD × 100(2)

MSF_SC_ = MSF − 0.5 × (SlD_F_ − SlD) (3)

SJL = MSF − MSW(4)

where SlD_F_: sleep duration on free days; SlD_W_: sleep duration on weekdays; SlD: average weekly sleep duration; TiB: time in bed; FD: number of free days; SlE: sleep efficiency; MSF: mid-point of the sleep phase on free days; MSW: mid-point of the sleep phase on weekdays; SJL: social jetlag; and MSF_SC_: mid-point of the sleep phase on free days, adjusted by the sleep debt accumulated on weekdays (chronotype).

#### 2.2.4. PSQI

To assess sleep quality, we used the Russian version of the PSQI [34]. This test consists of 19 questions related to sleep quality, including sleep latency, duration, efficiency, disturbance, use of sleep medication, and daytime sleepiness for a one-month period. Global PSQI scores range from 0 to 21 points. In our sample, the scores ranged from 0 to 16, with an overall group *M* (SD) of 6.4 (2.8). According to the test authors, a PSQI score of ≤5 indicates good-quality sleep, and a PSQI score of >5 indicates poor-quality sleep [29].

#### 2.2.5. ZSDS

Depression levels were assessed using the ZSDS test [28]. The ZSDS comprises 20 statements describing depression symptoms. The sum of raw ZSDS scores ranging from 20 to 80 was converted to ZSDS indices (ZSDSIs) varying from 25 to 100, as described by Zung [35] and Passik et al. [36]. The ZSDSIs were used to evaluate four levels of depression: I—no depression (ZSDSI ≤ 50); II—minimal to mild depression (ZSDSI 51–59); III—moderate to significant depression (ZSDSI 60–69); and IV—severe to extreme depression (ZSDSI ≥ 70). Cronbach’s α for this sample was 0.769.

#### 2.2.6. MT-Containing Foods Consumption

FMT intake was assessed using the modified food frequency questionnaire (FFQ). Each study participant was asked to choose from a list of products that, according to previous studies, contain MT. They were also asked to answer the following questions:

1. How often have you consumed these foods in the past month? Answer options: never, 1–2 times a month, 3–4 times a month, 2–3 times a week, 4–6 times a week, 1–2 times a day, 3–4 times a day, more than 4 times a day.
2. How many servings of these foods did you consume in one meal (this question was accompanied by a picture indicating the size of one serving and the product’s weight in grams)? Answer options: 0.5, 1, 2, 3, 4, or 5 servings.
3. What percentage of the foods above was eaten during dinner? The response options were 0, 25, 50, 75, or 100%.

These data were used to calculate FMT consumption per day (FMT_day_) and per dinner (FMT_dinner_) by multiplying the average number of MT-containing foods consumed per day and at dinner by the average MT content in those products (Table S1). The calculations are presented in Supplementary Materials.

### 2.3. Data and Statistical Analyses

We used SPSS version 20 (SPSS, Inc., Chicago, IL, USA) for statistical data analyses. Table 1 presents the continuous variables (mean, standard deviations, and estimates of normality of distribution) used in this study. The distribution of six variables (Age, BMI, WHtR, SlE, FMT_day_, and FMT_dinner_) differed from normal. Therefore, in further analyses, transformed indicators (Agec, BMI%, WHtRc, SlEc, FMT1_day_, and FMT1_dinner_) with a normal distribution were used (Table 1). Table 2 presents the categorical variables and codes.

A series of multiple regression analyses were performed in which the continuous values BMI%, WHtRc, MSFsc, SJL, SlD, SlEc, PSQI, ZSDSI, and GPA were used as dependent variables, and Agec, Sex (codes: 1—females; 2—males), FMT1_day_, FMT1_dinner_, city (codes: 1—Syktyvkar, 2—Kirov, 3—Tyumen), and mode of study (codes: 1—regular, 2—remote) were used as independent variables (predictors). A procedure of stepwise inclusion was used to determine predictors for the model. Only predictors with significant regression coefficients were included in the final model. The variance inflation factor was used to evaluate multicollinearity in the model, as described in Dormann et al. [37]. Predictors were excluded from the model if the variance inflation factor was ≥5.

A series of binary logistic regression analyses were performed using Ov/Ob (Codes: 0—BMIc 1 + 2, 1—BMIc 3 + 4), WHtRc (0—WHtR < 0.5, 1—WHtR ≥ 0.5), SJL (0—SJL < 1, 1—SJL ≥ 1), PSQIc (0—PSQI ≤ 5, 1—PSQI > 5), ZSDSIc (0—ZSDSI < 60, 1—ZSDSI ≥ 60) as dependent variables and Agec, Sex, FMT1_day_, FMT1_dinner_, city (codes: 1—Syktyvkar, 2—Kirov, 3—Tyumen), and mode of study (codes: 1—Regular, 2—Remote) were included as independent variables. A procedure of stepwise inclusion was used to determine the final set of predictors in the model. The goodness of fit was evaluated by the Hosmer–Lemeshow test and Omnibus tests of model coefficients.

Two analyses of covariance (ANCOVAs) were performed using tertiles of FMT1_day_ and FMT1_dinner_ as fixed factors; BMI%, WHtRc, SJL, MSFsc, SlD, SlEc, PSQI, ZSDSI, and GPA as dependent variables; and Agec, Sex (codes: 1—females; 2—males) city (codes: 1—Syktyvkar, 2—Kirov, 3—Tyumen), and mode of study (codes: 1—regular, 2—remote) as covariates. Eta-squared (*ɳ*^2^) was used to evaluate effect size.

## 3. Results

The mean dietary intake of FMT_day_ was *M* (SD) 2209.8 (4183.2) ng/day and FMT_dinner_ 897.8 (2536.8) ng/dinner (Table 3).

**Figure 1:**
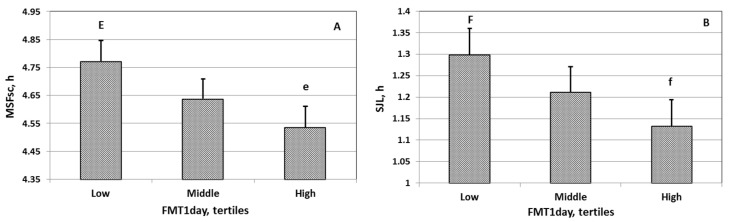
The association of FMT1_day_ with MSFsc (**A**) and SJL (**B**). The differences between the compared groups are significant E > e (*p* < 0.001), F > f (*p* < 0.05). Abbreviations: MSFsc: chronotype; SJL: social jetlag; FMTday: total daily food melatonin consumption; FMT1_day_ = *Ln*(FMT_day_); Error bars: SD.

Analysis of covariance showed a significant association between FMT_day_ and central adiposity, chronotype, social jetlag, sleep quality, and depression (Table 4, Figure 1A,B). A significant association between FMT_dinner_ and central adiposity was noted (Table 5). The indices characterizing central adiposity, chronotype, social jetlag, and depression were significantly lower in people consuming FMT_day_ more than 1651.1 ng/day (Table 3). The central adiposity index was significantly lower in people consuming FMT_dinner_ more than 577.3 ng/dinner (Table 3).

Students who consumed more FMT_day_ and FMT_dinner_ had lower central adiposity index values (Models 1 and 2, Table 6). Students who consumed more FMT_day_ had an earlier chronotype (Model 3, Table 6), less social jetlag (Model 4, Table 6), better sleep quality (Model 5, Table 6), and fewer depression symptoms (Model 6, Table 6).

The logistic regression analysis indicated that schoolchildren and students with higher FMT_day_ consumption did not show moderate/severe depression symptoms (Table 7).

## 4. Discussion

Our study is the first to show that total FMT intake is associated with sleep–wake rhythm and social jetlag in adolescents and young adults. Adolescents and young adults who consume more FMT-containing foods throughout the day have a less pronounced delay in sleep–wake rhythm phase and circadian misalignment. We found a direct association between FMT_day_ and MCTQ-derived “weekend mid-sleep phase adjusted for school-day sleep debt (MSFsc)” [7]. This indicator is a quantitative measure of an individual’s chronotype. Previously, MSFsc and dim light MT onset (DLMO) have been closely associated [38,39], representing a reliable marker of the endogenous rhythm in human CS [5]. In addition, we noted an inverse association between FMT_day_ and SJL, a quantitative measure of circadian misalignment [6].

The data obtained indicate that FMT consumption is one of the ways to prevent SJL and, consequently, circadian-misalignment-related problems in adolescents, such as low academic performance [10], depression [12], and obesity [13]. This concept is supported by the association between FMT and anthropometric measures (central adiposity) as well as psychoemotional indicators (depression). This conclusion has practical importance since circadian misalignment has become widespread among students. In different countries, social jetlag detection rates widely vary, from 40.1% in Japan [40] to 86.4% in Russia [8].

Our study also showed that schoolchildren and university students who consume more FMT during the day have higher sleep quality. These data are consistent with previously published data on FMT’s positive effect on sleep function [19,20,21,22,23]. In particular, MT-rich foods for dinner and breakfast have previously been shown to increase sleep duration and efficiency [19,21,22] as well as reduce sleep latency [19] in adults and the elderly. One study [22] found that drinking cherry juice concentrate with high MT content in the morning and evening for a week increases the amplitude and mesor, but not the phase of the 24-h rhythm of MT metabolite excretion in the urine. The authors also noted an increase in sleep duration and efficiency, as assessed by actimetry. Cherry juice concentrate similarly affected the sleep quality of 65-year-olds in another study [20]. In young adults with low self-reported sleep quality, consuming two kiwifruit an hour before bedtime for four weeks increased PSQI-derived total sleep duration and efficiency [21].

It should be noted that not all study results can be logically explained within our hypothesis regarding FMT’s chronobiotic effects. The close relationship between FMT_day_ and the studied indicators, compared with FMT_dinner_, does not fit with the framework of this concept. The weak association of FMT_dinner_ with sleep-wake rhythm characteristics may be due to the presence of substances in food that prevent FMT’s action. However, analysis of such substances was not performed in the present study. It can be speculated that food containing an effective FMT dose also contains an excess amount of carbohydrates, fats, etc., which can interfere with FMT’s positive effects. Sleep quality is adversely affected by excessive food intake before bedtime [41], and excessive fat content in one’s daily diet negatively affects CS function [42]. Eating a high-calorie meal for dinner may also delay the sleep–wake rhythm phase [43].

A more pronounced chronobiotic effect of FMT_day_, compared with FMT_dinner_, could be explained by the fact that adolescents and young adults consume the bulk of their daily diet in the afternoon. Circadian misalignment causes significant changes in eating behavior, such as skipping breakfast [44] and refusing a full lunch during classes [45]. As previously demonstrated, the sleep–wake rhythm phase shifted earlier when exogenous MT was administered in the afternoon, even 11 h before the sleep midpoint [46].

At a dose of 1300 ng/day of FMT_day_, we observed a significant change in most of the parameters studied. This dosage is significantly less than the minimum dose of exogenous MT (0.3–0.5 mg), at which chronobiotic effects were previously noted [46,47]. Ingesting 0.3 mg of MT in the second half of the day has been found to shift the sleep–wake rhythm phase to an earlier time of day [46]. In this regard, we should note that our methodology can only be characterized as semi-quantitative, providing a rough estimate of FMT consumption. We did not evaluate the dietary intake of tryptophan, a precursor to serotonin and MT biosynthesis. The consumption of cereals enriched with tryptophan (60 mg) for dinner and breakfast leads to an increase in urinary excretion of MT metabolites, positively affecting sleep function and reducing depression risk in the elderly [48]. Furthermore, it cannot be ruled out that the FMT dose affecting CS function during chronic consumption is significantly lower than with short-term exogenous MT administration. Nagata et al. [27], who carefully estimated total dietary FMT intake, found even lower FMT_day_ values of 29.8–32.3 ng/day. At the same time, the authors noted a significant inverse relationship between FMT_day_ and total mortality risk in a large sample of Japanese residents > 35 years old (about 30,000 people).

Our work has several strengths and perspectives. An inverse relationship was first noted between FMT and social jetlag and indicators closely related to circadian misalignments, such as obesity and depression. We suggested that FMT_day_ and FMT_dinner_ can be used as integrated indicators of MT-containing foods consumption to help develop regimens and diets to prevent the negative consequences of circadian desynchrony. At the same time, this work has limitations. It should be noted that FFQ validation is required to determine food MT intake. The FMT_day_ and FMT_dinner_ indicators were calculated based on the literature data. MT content in individual products, according to different authors, widely varies depending on many factors. We used a semi-quantitative scale to reduce the effects of a wide range of factors on MT content in food. The study did not take into account the influence of lifestyle factors, such as the level of physical activity, caloric intake, coffee, and alcohol and nicotine consumption. This appeared to have reduced the accuracy of the analysis of the relationship between FMT and indicators characterizing human CS. Respondents were asked to fill out a questionnaire in which they indicated the frequency of consumption of melatonin-containing foods over the past month. It should be taken into account that this approach reduced the reliability of the collected data due to possible inaccuracies in the respondents’ recollection of their diet. Most of the study participants (72.8%) were women. In this study, we did not take into account the influence of another factor, the state of the reproductive function. It is known that there are significant changes in sleep function and psychoemotional state in women during the menstrual cycle, associated with changes in the production of sex hormones [49] and melatonin [50]. In the future, it will be necessary to conduct a special study to take into account the influence of the state of reproductive function on the association among studied indicators. The cross-sectional design of our study did not allow us to judge causal relationships between the studied indicators.

## 5. Conclusions

As a result of the study, it was found that higher consumption of melatonin-containing foods per day is associated with early chronotype and less social jetlag, better sleep quality and lower levels of depression, and central adiposity. Higher consumption of melatonin-containing foods for dinner is associated with a lower risk of central adiposity. The findings indicate that the potential chronobiotic effect of the diet may be partly due to dietary melatonin.

## Figures and Tables

**Table 1:** Descriptive statistics of continuous variables used in the study.

Parameters, Units	Abbreviation	Mean	*SD*	*S*	*K*
Age, years	Age	19.91	4.11	4.02	20.50
	^a^ Agec	4.48	2.06	−0.38	0.08
Body mass index, kg/m^2^	BMI	21.72	3.68	1.40	3.18
	^b^ BMI%	48.06	24.73	0.07	−0.51
Waist to height ratio, cm	WHtR	0.42	0.06	0.63	3.01
	^c^ WHtRc	0.37	0.07	0.41	−0.73
Social jetlag, h	SJL	1.21	1.26	0.65	0.72
Chronotype, h	MSF_sc_	4.65	1.52	0.36	0.80
Sleep duration, h	SlD	7.09	1.39	−0.12	0.33
Sleep efficiency, %	SlE	87.36	9.00	−1.66	3.99
	^d^ SlEc	82.73	8.59	−0.45	0.32
Sleep quality, scores	PSQI	6.31	2.80	0.54	0.16
Depression, scores	ZSDSI	47.36	12.25	−0.50	0.22
Academic performance, scores	GPA	4.30	0.51	−0.38	−0.71
Food MT consumption for day, ng	FMT_day_	2209.76	4183.19	7.49	81.68
	^e^ FMT1_day_	7.01	1.13	0.21	0.32
Food MT consumption for dinner, ng	FMT_dinner_	897.75	2536.80	10.86	153.43
	^f^ FMT1_dinner_	5.68	1.50	−0.14	0.49

*SD*: standard deviation; *S*: skewness; *K*: kurtosis; ^a^ Agec = 1/exp(age); ^b^ BMI%: BMI percentiles (see Materials and Methods for details); ^c^ WHtRc: categories were defined as described in Table 2; ^d^ SlEc: categories were defined as described in Table 2; ^e^ FMT1_day_ = *Ln*(FMT_day_); ^f^ FMT1_dinner_ = *Ln*(FMT_dinner_).

**Table 2:** Descriptive statistics of categorical variables used in the study.

Parameter	Categories	*N*	%
City	Syktyvkar	280	21.92
	Kirov	263	20.60
	Tyumen	734	57.48
Study mode	Regular	928	72.67
	Remote	349	27.33
Sex	Female	930	72.83
	Male	347	27.17
WHtRc, categories	≤0.39	483	37.82
	0.40–0.49	653	51.14
	≥0.5	141	11.04
WHtRc1 categories	<0.5	1020	88.96
	≥0.5	120	11.04
BMIc, categories	Underweight/Normal weight	1140	86.45
	Overweight/Obese	173	13.55
SlEc, categories	≤79	215	16.83
	80–89	434	33.99
	90–99	482	37.74
	100	146	11.43
PSQIc, categories	≤5	540	42.29
	>5	737	57.71
ZSCSIc, categories	No to minimal depression	1046	81.91
	Moderate to severe depression	231	18.09

**Table 3:** Descriptive statistics of FMT_day_ and FMT_dinner_.

Parameter	Tertiles	*M*	*SD*	Min	Max
FMT_day_	1 (Low)	277.49	154.25	10.92	646.80
	2 (Middle)	1063.84	279.28	647.29	1650.39
	3 (High)	5194.97	6232.46	**1651.09**	67,614.85
FMT1_day_	1 (Low)	5.82	0.55	2.39	6.47
	2 (Middle)	6.93	0.26	6.47	7.41
	3 (High)	8.27	0.65	7.41	11.12
FMT_dinner_	1 (Low)	77.28	43.09	1.25	156.54
	2 (Middle)	310.78	109.71	157.01	577.02
	3 (High)	2300.28	4030.83	**577.29**	41,677.00
FMT1_dinner_	1 (Low)	4.08	0.90	0.22	5.06
	2 (Middle)	5.69	0.35	5.06	6.37
	3 (High)	7.30	0.79	6.37	10.64

FMT1_day_ = *Ln*(FMT_day_); FMT1_dinner_ = *Ln*(FMT_dinner_). Values marked in bold are significant; data presented as *M* (SD).

**Table 4:** Association of FMT1_day_ with anthropometric, sleep and psychoemotional characteristics, and academic performance.

Dependent Variables	Tertiles of FMT_day_			*F*	*P*	*ɳ* ^2^
	1 (Low) ^1^	2 (Middle)	3 (High)			
BMI%	48.41 (24.46)	47.37 (24.99)	48.35 (24.74)	0.422	0.656	0.001
WHtRc, cm	0.42 (0.06)	0.42 (0.07)	0.41 (0.07)	**3.024**	**0.049**	**0.005**
SlD, h	7.17 (1.40)	7.11 (1.37)	7.01 (1.40)	1.388	0.250	0.002
PSQI, scores	6.63 (2.83) ^B^	6.25 (2.75)	6.01 (2.80) ^b^	**3.326**	**0.036**	**0.005**
MSFsc, h	4.77 (1.54) ^C^	4.64 (1.46)	4.54 (1.54) ^c^	**5.373**	**0.005**	**0.008**
SJL, h	1.30 (1.28) ^A^	1.21 (1.22)	1.13 (1.27) ^a^	**3.038**	**0.048**	**0.005**
ZSDSI, scores	48.87 (12.32) ^D^	47.95 (12.17) ^C^	45.19 (11.93) ^c,d^	**7.620**	**0.001**	**0.012**
GPA, scores	4.24 (0.52) ^c^	4.37 (0.50) ^C^	4.31 (0.50)	2.848	0.058	0.005

FMT_day_: values of food melatonin consumption for day as described in Table 1; ^1^ threshold values of tertiles presented in Table 3; BMI%: body mass index, percentiles; WHtR: waist to height ratio; SlD: sleep duration; PSQI: sleep quality, global scores; MSFsc: chronotype; SJL: social jetlag; ZSDSI: depression, scores; GPA: academic performance; ANCOVAs were performed using variables marked in the left column as dependent variables, “FMT_day_” as fixed factor and “agec” and “sex” (codes: 1—females, 2—males), “city” (codes: 1—Syktyvkar, 2—Kirov, 3—Tyumen), and “mode of study” (codes: 1—regular, 2—remote) as covariates; *F*: Fisher test; *P*: significance of *F*-test; *ɳ*^2^: effect size; values marked in bold are significant; data presented as *M* (SD); post hoc comparisons ^A^ > ^a^: *p* < 0.05; ^B^ > ^b^: *p* < 0.01; ^C^ > ^c^: *p* < 0.001 ^D^ > ^d^: *p* < 0.0001.

**Table 5:** Association of FMT1_dinner_ with anthropometric, sleep, psychoemotional characteristics, and academic performance.

Dependent Variables	Tertiles of FMT_dinner_			*F*	*P*	*ɳ* ^2^
	1 (Low) ^1^	2 (Middle)	3 (High)			
BMI%	48.59 (25.28)	48.17 (23.76)	47.65 (25.16)	0.609	0.544	0.001
WHtRc	0.43 (0.06) ^A^	0.42 (0.06)	0.41 (0.07) ^a^	4.203	0.015	0.008
SlD	7.15 (1.36)	7.14 (1.35)	6.99 (1.43)	1.175	0.309	0.002
PSQI	6.41 (2.82)	6.29 (2.72)	6.19 (2.76)	0.172	0.842	0.000
MSFsc	4.67 (1.51)	4.65 (1.52)	4.63 (1.54)	0.800	0.450	0.001
SJL	1.24 (1.22)	1.23 (1.29)	1.16 (1.26)	0.370	0.690	0.001
ZSDSI	48.66 (12.57) ^B^	47.21 (11.83)	46.30 (11.88) ^b^	2.228	0.108	0.004
GPA	4.26 (0.51) ^a^	4.34 (0.51) ^A^	4.31 (0.51)	2.434	0.088	0.004

FMT_dinner_: values of food melatonin consumption for dinner as described in Table 1; ^1^ threshold values of tertiles presented in Table 3; the rest of the abbreviations and indices are as described in Table 4; data presented as *M* (SD). Post hoc comparisons ^A^ > ^a^: *p* < 0.05; ^B^ > ^b^: *p* < 0.01.

**Table 6:** Results of multiple regression analyses ^a^.

#	Dependent Variable	Predictors	*B*	*β*	*R* ^2^	∆*R*^2^	*P*	*VIF*
1	WHtRc	Agec	0.003	0.180	0.034	0.034	0.000	1.002
		Sex	0.022	0.144	0.053	0.018	0.000	1.011
		FMT1_day_	−0.005	−0.082	0.059	0.007	0.005	1.012
2	WHtRc	Agec	0.003	0.180	0.033	0.033	0.000	1.000
		Sex	0.022	0.144	0.052	0.019	0.000	1.005
		FMT1_dinner_	−0.003	−0.083	0.059	0.007	0.005	1.005
3	MSFsc	Agec	−0.033	−0.091	0.008	0.008	0.001	1.000
		FMT1_day_	−0.112	−0.088	0.016	0.008	0.002	1.000
4	SJL	Agec	−0.026	−0.084	0.007	0.007	0.003	1.000
		FMT1_day_	−0.104	−0.068	0.012	0.005	0.016	1.000
5	PSQI	Sex	−1.155	−0.183	0.036	0.036	0.000	1.013
		FMT1_day_	−0.132	−0.056	0.039	0.003	0.043	1.013
6	ZSDSI	Sex	−6.359	−0.232	0.060	0.060	0.000	1.012
		FMT1_day_	−1.131	−0.111	0.072	0.012	0.000	1.012

WHtRc: waist to height ratio, categorical; MSFsc: chronotype; SJL: social jetlag; PSQI: sleep quality, global scores; ZSDSI: depression, scores; ^a^ each model included indicators presented in Table 1 as dependent variables and “FMT1_day_”, “FMT1_dinner_”, “agec“, “sex“ (codes: 1—females; 2—males), “city“ (codes: 1—Syktyvkar, 2—Kirov, 3—Tyumen), and “mode of study” (codes: 1—regular, 2—remote), in the models 3–6 “BMIc“ (codes: 1—underweight; 2—normal weight; 3—overweight; 4—obese) as predictors, but only significant variables were included in the final model; *B*: non-standardized regression coefficient; *β*: standardized regression coefficient; *P*: Bonferroni-corrected significance of *B; R*^2^: total variance accounted for predictors at their stepwise inclusion in the model; Δ*R*^2^: portion of the variance accounted for by separate predictors in the model; *VIF*: variation inflation factor.

**Table 7:** Results of logistic regression analyses ^a^.

Dependent Variable	Predictors	*B*	*OR*	*95% CI*		*P ^b^*	Omnibus Test		Hosmer-Lemeshov Test	
							*χ^2^*	*P*	*χ^2^*	*P*
ZSDSIc	Sex	−1.058	0.374	0.230	0.525	0.000	41.264	0.000	2.641	0.955
	FMT1_day_	−0.180	0.835	0.736	0.948	0.005				

BMIc: body mass index, categorical (codes: 0—underweight/normal weight, 1—overweight/obesity); ZSDSIc: depression (codes: 0—no/light depression, 1—moderate/severe depression); ^a^ a series of logistic regression analyses were performed using the indicators presented in Table 3 specified as dependent variables, while “agec“, “sex“ (codes: 1—female; 2—male) “city“ (codes: 1—Syktyvkar, 2—Kirov, 3—Tyumen), “mode of study“ (codes: 1—regular, 2—remote), and indicator “BMIc“ (codes: 1—underweight; 2—normal weight; 3—overweight; 4—obese) in the Model 2 were specified as independent variables; the code “0” was used in the models as the comparison group. *B*: regression coefficient; *OR*: odds ratio; *CI*: confidence interval; *^b^ P*: Bonferroni-corrected significance of the regression coefficient; Omnibus and Hosmer–Lemeshow test: goodness of fit tests used for the models.

## Data Availability

The data of this study are available on request from the corresponding author.

## Supplementary Materials

The following supporting information can be downloaded at: https://www.mdpi.com/article/10.3390/nu15153302/s1, Figure S1: Picture indicating portions size; Table S1: FMT concentration in food products, ng/g fresh weight; Table S2: Rounded values of FMT content in 5 product categories; Table S3: Frequency of product consumption; Table S4: the sizes of food portions in grams; Table S5: Examples of a list of products containing threshold amounts of FMT corresponding to the lower threshold of the 3rd tertile of FMT_day_ and FMT_dinner_. References [15,16,17,20,22,51,52,53,54,55,56,57,58,59,60,61,62,63,64,65,66,67,68,69,70,71,72,73,74,75,76,77,78,79,80,81,82,83,84,85,86,87] are cited in the supplementary materials.
