# Supplementary material for: The Association between Melatonin-Containing Foods Consumption and Students’ Sleep–Wake Rhythm, Psychoemotional, and Anthropometric Characteristics: A Semi-Quantitative Analysis and Hypothetical Application
Source: Nutrients. 2023 Jul 25;15(15):3302. doi: 10.3390/nu15153302 (PMC10420797; doi:10.3390/nu15153302)
Supplement: Supplementary file 1 [file nutrients-15-03302-s001.zip › nutrients-2471316-supplementary.pdf]

Assessment of food melatonin consumption

A modified food frequency questionnaire was used to assess the consumption of FMT with food. The questionnaire included only products that, according to the literature, contain MT as well as those products that are used for food by residents of Russia.

1. To calculate the FMT content in food products, literature data were used (1-42). An analysis of the literature has shown that the content of FMT varies widely, both between different products and within the same product, depending on the method of analysis, plant variety, and method of preparation of the product. Therefore, we extracted from this literature (1-42) all references to the FMT content in certain products (**Table S1**), calculated the median of the FMT content in each product, and grouped the products into 5 categories, as shown in Tables S1 and S2. Rounded FMT concentration values (**C_{FMT}**; **Table S2**) were used to calculate FMT consumption by study participants.

Table S1. FMT concentration in food products, ng/g fresh weight[1]

	Product	FMT concentration according to literature data																				Median
1	White mustard	189																				189.00
	Black mustard	129																				129.00
	Curcuma	120																				120.00
	Mulberry	90																				90.00
2	Soybean	56.5	1.89																			29.20
	Rice	212.0	182.0	73.81	47.83	42.95	38.46	31.99	28.33	27.61	5.2	2.7	1.9	1.5	1.0	1.0						28.33
	Apple	134	71	67.6	23	20	15.3	10	5	0.48	0.05	0.16										15.30
3	Strawberry	11.3	11.26	11	8.5	5.58	5.5	4.2	4	1.4	0.14	0.12	0.01									4.85
	Tomato	114.5	50.1	23.87	18.13	17.1	14.2	14	13.6	8.2	8	7.73	7.5	4.45	4.1	3.4	2.5	1.2	1.07	0.62	0.5	
		0.3	0.17	0.11	0.03	0.016																4.45
	Salmon	3.7																				3.70
	Pomegranate	5.5	5.5	0.54	0.17																	3.02
	Cherry	124.7	120	20	19.5	18	14	13.46	12.3	8	2.9	2.06	0.22	0.22	0.12	0.06	0.05	0.03	0.01	0.006	0.006	2,50
	Pork	2.5																				2.50
	Beef	2.1																				2.10
	Almond	39	2.6	1.05	0.12																	1.83
	Oat	1.8																				1.80
	Walnut	3.5	3.5	1.9	1.77	1.37	1.02	0.14														1.77
	Chicken	2.3	1.1																			1.70
	Corn	1.9	1.88	1.4	1.37																	1.64
	Lamb	1.6																				1,60
	Egg	1.54																				1.54
	Apple juice	1.45	1.4																			1.43
	Ginger	1.42																				1,42
	Orange	20	2.49	0.15	0.15																	1,32
	Grape	18	18	3	1.5	1.2	1.2	1.2	1	0.97	0.97	0.87	0.63	0.6	0.42	0.33	0.26	0.14	0.03	0.005		0.92
	Mango	0.7	0.70	0.26																		0.70
	White radish	0.66																				0.66
	Peanut	39.4	0.6	0.1																		0.60
	Pearl barley	0.87	0.58	0.38																		0.58
	Sunflower	67.5	29	0.2	0.2	0.55																0.55
	Grape juice	0.5																				0.50
4	Mango juice	0.38																				0.38
	Pineapple	0.36	0.302	0.302	0.3	0.28	0.04															0.30
	Banana	0.66	0.47	0.47	0.09	0.01	0.005															0.28
	Carrot	0.49	0.06																			0.28
	Papaya	0.24	0.24																			0.24
	Cabbage	0.31	0.11																			0.21
	Pineapple juice	0.20																				0.20
	Orange juice	3.15	0.185	0.15	0.096																	0.17
	Chinese cabbage	0.11																				0.11
	Olive oil	0.119	0.108	0.108	0.107	0.098	0.095	0.089	0.075	0.071	0.053	0.05										0.10
	Onion	0.3	0.09	0.03																		0.09
	Cucumber	0.59	0.09	0.086	0.025																	0.09
	Welsh onion	0.09																				0.09
	Sunflower oil	0.12	0.08	0.07	0.06	0.05	0.05	0.03														0.06
5	Kiwifruit	0.02																				0.02
	Cow milk	0.014																				0.01
	Beetroot	0.002																				0.002

[1]Data on concentration of FMT in products were extracted from publications 1-42. The table includes data on the content of FMT in the tissues/organs of plants commonly used for food. The table includes data on the content of FMT in seeds and other bulk products in terms of grams of fresh and/or dry matter. The FMT content in beverages is expressed in ng/ml.

Table S2. Rounded values of FMT content in 5 product categories[1]

Food product categories according to C_{FMT}	C_{FMT}, ng/g FW
1. High	100
2. Above average	10
3. Average	1
4. Below average	0.1
5. Low	0.01

[1] The distribution of products by category is shown in Table 1s.

2. In the **Table S3**, left column the options for answering the question about the frequency of product consumption are presented, in the right – the corresponding conversion coefficients of product consumption per day (**k**).

Table S3. Frequency of product consumption

1. How often (times) have You consumed the specified products?	Times a day (**k**)
Never	0
1-2 times a month	0.05
3-4 times a month	0.12
2-3 times a week	0.36
4-6 times a week	0.71
1-2 times a day	1.50
3-4 times a day	3.50
>4 times a day	5

4. It is suggested to select products from a list in which each item is illustrated with picture indicating the size of portions (**Figure S1**).

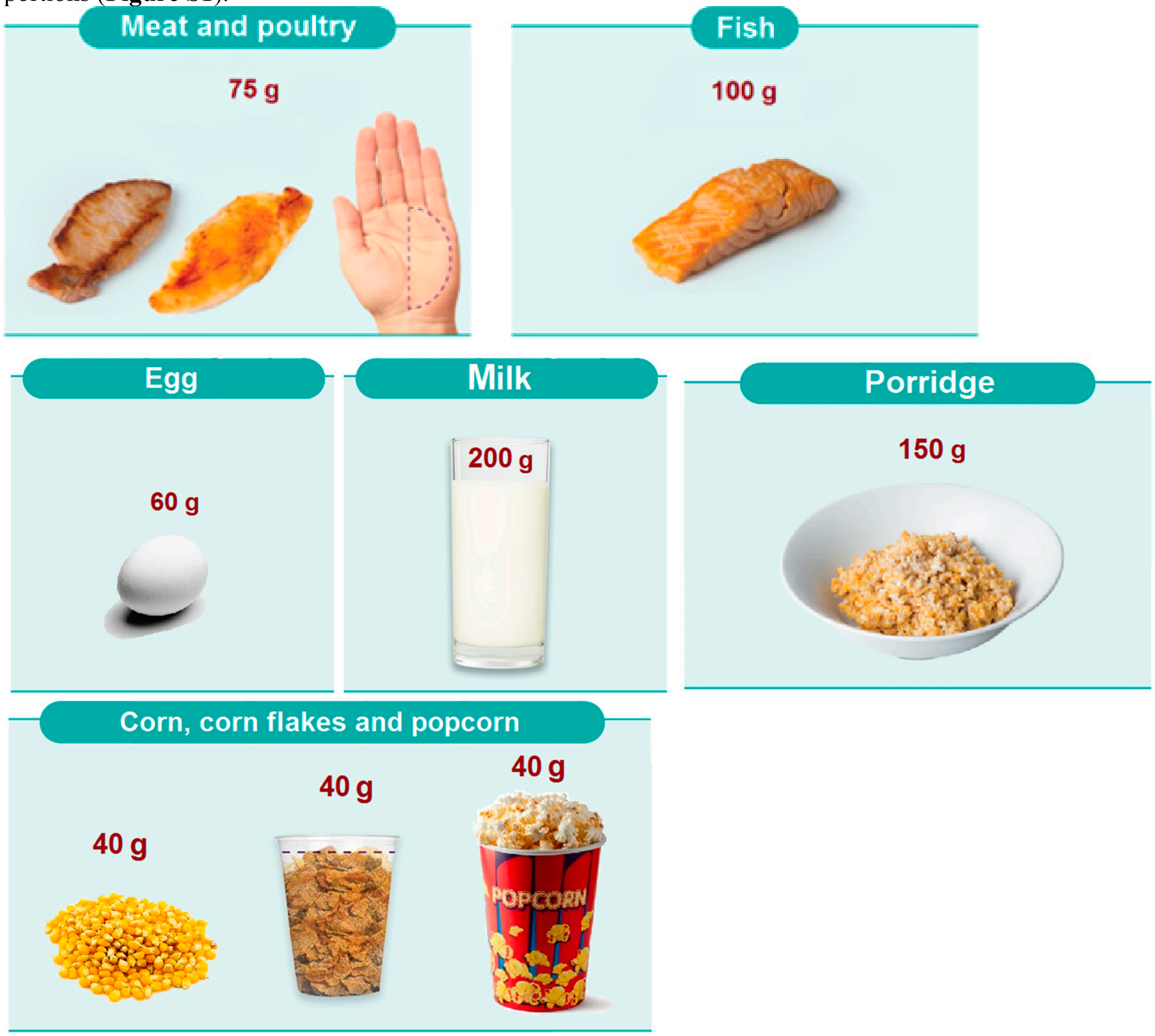

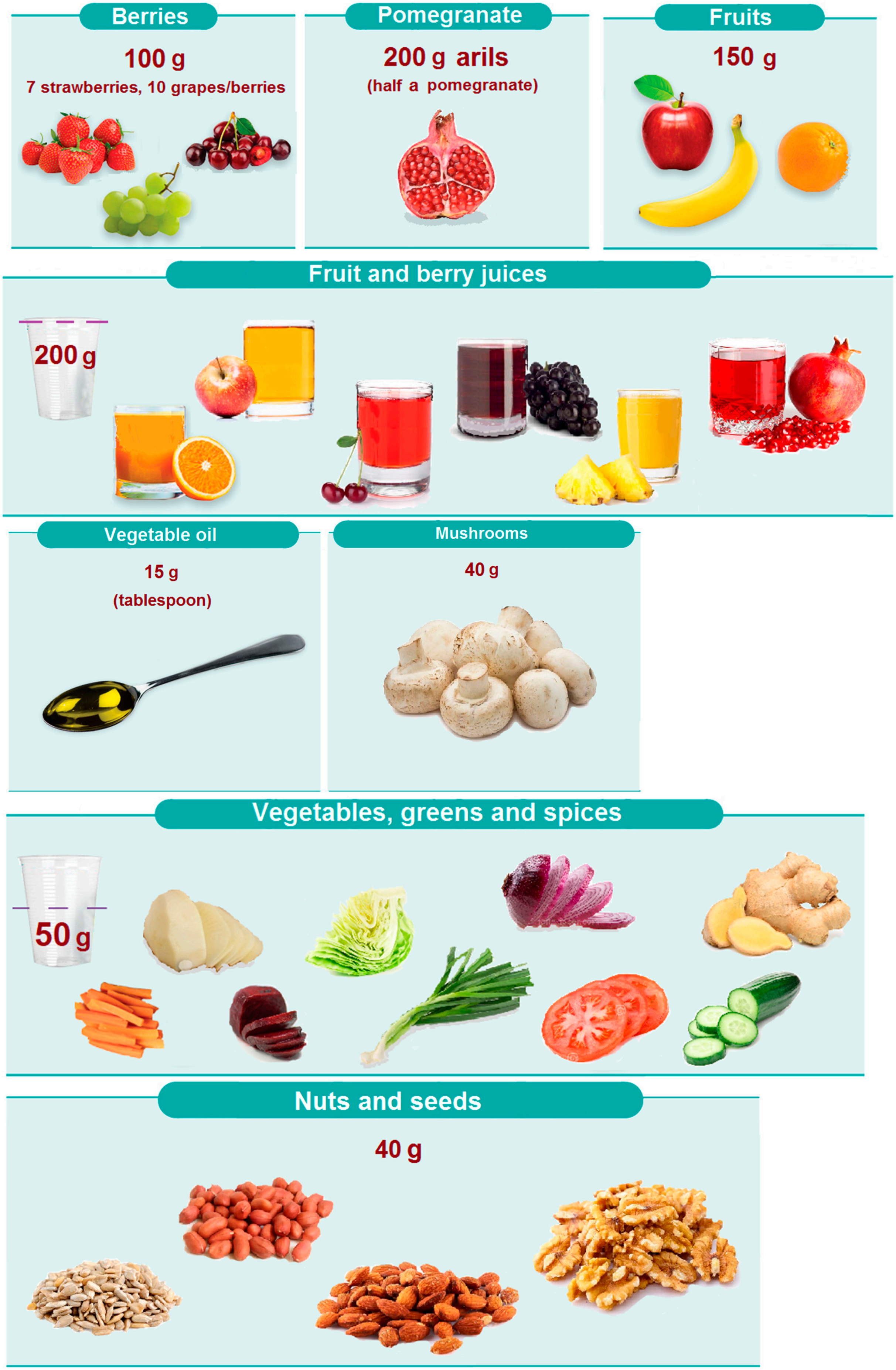

Figure S1. Illustration of portion sizes of foods.

3. Table S4 shows the sizes of food portions in grams (**M**).

Table S4. Serving sizes in grams

Product	**M**, g
Meat	75
Fish	100
Egg	60
Milk	200
Porridge	150
Corn	40
Berries	100
Pomegranate	200
Fruits	150
Juice	200
Oil	14
Vegetables	50
Nuts	40

4. Each study participant was asked to indicate the number of servings consumed at one meal (**p**):

Response options: **p** = 0.5; 1; 2; 3; 4; 5 and more.

5. Each participant of the study was asked to indicate the part of the daily portion of the product that he/she eats for dinner (**d**):

Response options: **d** = 0; 10; 20; 30 … 100%

6. Formulas (**1s-4s**) were used to calculate the daily consumption of FMT (FMT_{day}) and for dinner (FMT_{dinner}):

$$\mathbf{a_n = M \cdot k \cdot p \cdot C_{FMTan}} \quad \text{(S1)}$$

$$\mathbf{FMT_{day} = \sum (a_1 + a_2 + \ldots a_n)} \quad \text{(S2)}$$

$$\mathbf{b_n = a_n \cdot d_n / 100} \quad \text{(S3)}$$

$$\mathbf{FMT_{dinner} = \sum (b_1 + b_2 + \ldots b_n)} \quad \text{(S4)}$$

were $\mathbf{a_n}$ – the amount of FMT consumed per day with one product, ng; **M** – serving size, g (**Table 4s**); **k** – the frequency of consumption of the product per day (Table 3s); **p** – the number of servings of the product consumed at a time; $\mathbf{C_{FMTan}}$ – the concentration of FMT in this product (Tables 1s and 2s); $\mathbf{a_1}$**;** $\mathbf{a_2}$**; ...**$\mathbf{a_n}$ – products from Table 1s that the study participant noted in his questionnaire; $\mathbf{b_n}$ **-** the amount of FMT consumed for dinner with one product; $\mathbf{d_n}$ – part of the daily consumption of the product that the study participant ate for dinner, %; $\mathbf{b_1}$**;** $\mathbf{b_2}$**; ...**$\mathbf{b_n}$ – products from Table 1s that the study participant consumed for dinner, ng.

Table S5 presents calculations and examples of a list of products that contain amounts of FMT corresponding to the lower threshold of the 3rd tertile of FMT_{day} and FMT_{dinner}.

Table S5. Examples of a list of products containing threshold amounts of FMT corresponding to the lower threshold of the 3rd tertile of FMT_{day} and FMT_{dinner}

#	Product	C_{FMT}, ng/g	M, g	k	p	FMT, ng
**1**	Rice	10	150	1	0,5	750
	Tomato	1	50	1	1	50
	Salmon	1	100	1	1	100
	Cherry	1	100	1	1	100
	Walnut	1	40	1	1	40
	Chicken	1	75	1	1	75
	Egg	1	60	1	1	60
	Apple juice	1	200	1	1	200
	Orange	1	150	1	1	150
	Grape	1	100	1	1	100
	Cabbage	0.1	50	1	1	5
	Orange juice	0.1	200	1	1	20
	Sunflower oil	0.1	14	1	1	1.4
	**Total FMT, ng:**					**1651.4**
**2**	Tomato	1	50	1	1	50
	Pomegranate	1	200	1	1	200
	Chicken	1	75	1	1	75
	Egg	1	60	1	1	60
	Orange	1	150	1	1	150
	Banana	0.1	150	1	1	15
	Cabbage	0.1	50	1	1	5
	Orange juice	0.1	200	1	1	20
	Olive oil	0.1	14	1	1	1.4
	Sunflower oil	0.1	14	1	1	1.4
	**Total FMT, ng:**					**577.8**

Abbreviations: $\mathbf{C_{FMT}}$ – FMT concentration in food product, ng/g; **M** – serving size, g; **k** – the frequency of consumption of the product per day; **p** – the number of servings of the product consumed at a time; **FMT** – consumption of FMT with the product, ng; **Total FMT** – total consumption of FMT, ng.

References

1. Dubbels R, Reiter RJ, Klenke E, et al. Melatonin in edible plants identified by radioimmunoassay and by high performance liquid chromatography-mass spectrometry. J Pineal Res 1995;18(1):28-31. doi:10.1111/j.1600-079x.1995.tb00136.x
2. Hattori A, Migitaka H, Iigo M, Itoh M, et al. Identification of melatonin in plants and its effects on plasma melatonin levels and binding to melatonin receptors in vertebrates. Biochem Mol Biol Int. 1995;35(3):627-634.
3. Manchester LC, Tan DX, Reiter RJ, et al. High levels of melatonin in the seeds of edible plants: possible function in germ tissue protection. Life Sci 2000;10;67(25):3023-3029. doi:10.1016/s0024-3205(00)00896-1.
4. Burkhardt S, Tan DX, Manchester LC, et al. Detection and quantification of the antioxidant melatonin in Montmorency and Balaton tart cherries (Prunus cerasus). J Agric Food Chem 2001;49(10):4898-4902. doi:10.1021/jf010321+
5. Badria FA. Melatonin, serotonin, and tryptamine in some egyptian food and medicinal plants. J Med Food 2002;5(3):153-157. doi:10.1089/10966200260398189
6. Hardeland R, Pandi-Perumal SR. Melatonin, a potent agent in antioxidative defense: actions as a natural food constituent, gastrointestinal factor, drug and prodrug. Nutr Metab (Lond) 2005;2:22. doi:10.1186/1743-7075-2-22
7. Reiter RJ, Manchester LC, Tan DX. Melatonin in walnuts: influence on levels of melatonin and total antioxidant capacity of blood. Nutrition 2005;21(9):920-924. doi:10.1016/j.nut.2005.02.005
8. Pape C, Lüning K. Quantification of melatonin in phototrophic organisms. J Pineal Res 2006;41(2):157-165. doi:10.1111/j.1600-079X.2006.00348.x
9. Iriti M, Rossoni M, Faoro F. Melatonin content in grape: myth or panacea? J Sci Food Agric 2006;86(10): 1432-1438. doi:10.1002/jsfa.2537

10. de la Puerta C, Carrascosa-Salmoral MP, García-Luna PP, et al. Melatonin is a phytochemical in olive oil. Food Chemistry 2007;104(2):609-612. doi:10.1016/j.foodchem.2006.12.010
11. Arnao MB, Hernández-Ruiz J. Melatonin promotes adventitious- and lateral root regeneration in etiolated hypocotyls of Lupinus albus L. J Pineal Res 2007;42(2):147-152. doi:10.1111/j.1600-079X.2006.00396.x
12. González-Gómez D, Lozano M, Fernández-León MF, et al. Detection and quantification of melatonin and serotonin in eight sweet cherry cultivars (Prunus avium L.). Eur Food Res Tech 2009;229:223–229. doi:10.1007/s00217-009-1042-z
13. Kirakosyan A, Seymour EM, Llanes DEU, et al. Chemical profile and antioxidant capacities of tart cherry products. Food Chem 2009;115(1):20-25. doi:10.1016/j.foodchem.2008.11.042
14. Pigeon WR, Carr M, Gorman C, Perlis ML. Effects of a tart cherry juice beverage on the sleep of older adults with insomnia: a pilot study. J Med Food 2010;13(3):579-583. doi:10.1089/jmf.2009.0096
15. Okazaki M, Ezura H. Profiling of melatonin in the model tomato (Solanum lycopersicum L.) cultivar Micro-Tom. J Pineal Res 2009;46(3):338-343. doi:10.1111/j.1600-079X.2009.00668.x
16. Kim SJ, Cho MH. Melatonin and polyphenol contents in some edible sprouts (alfalfa, chicory, rape, red kale and sunflower). Prev Nutr Food Sci 2011;16(2):184-188. doi:10.3746/jfn.2011.16.2.184
17. Rodriguez-Naranjo MI, Gil-Izquierdo A, Troncoso AM, et al. Melatonin: a new bioactive compound in wine. J Food Compost Anal 2011;24(4-5):603-608. doi:10.1016/j.jfca.2010.12.009
18. Stürtz M, Cerezo AB, Cantos-Villar E, Garcia-Parrilla MC. Determination of the melatonin content of different varieties of tomatoes (Lycopersicon esculentum) and strawberries (Fragariaananassa). Food Chem 2011;127(3):1329-1334. doi:10.1016/j.foodchem.2011.01.093
19. Howatson G, Bell PG, Tallent J, et al. Effect of tart cherry juice (Prunus cerasus) on melatonin levels and enhanced sleep quality. Eur J Nutr 2012;51(8):909-916. doi:10.1007/s00394-011-0263-7
20. Mercolini L, Mandrioli R, Raggi MA. Content of melatonin and other antioxidants in grape-related foodstuffs: measurement using a MEPS-HPLC-F method. J Pineal Res 2012;53(1):21-28. doi:10.1111/j.1600-079X.2011.00967.x
21. Johns NP, Johns J, Porasuphatana S, et al. Dietary intake of melatonin from tropical fruit altered urinary excretion of 6-sulfatoxymelatonin in healthy volunteers. J Agric Food Chem 2013;61(4):913-919. doi:10.1021/jf300359a
22. Zhao Y, Tan DX, Lei Q, et al. Melatonin and its potential biological functions in the fruits of sweet cherry. J Pineal Res 2013;55(1):79-88. doi:10.1111/jpi.12044
23. Sae-Teaw M, Johns J, Johns NP, Subongkot S. Serum melatonin levels and antioxidant capacities after consumption of pineapple, orange, or banana by healthy male volunteers. J Pineal Res 2013;55(1):58-64. doi:10.1111/jpi.12025
24. Lei Q, Wang L, Tan DX, et al. Identification of genes for melatonin synthetic enzymes in 'Red Fuji' apple (Malus domestica Borkh.cv.Red) and their expression and melatonin production during fruit development. J Pineal Res 2013;55(4):443-451. doi:10.1111/jpi.12096
25. Fernández-Pachón MS, Medina S, Herrero-Martín G, et al. Alcoholic fermentation induces melatonin synthesis in orange juice. J Pineal Res 2014;56(1):31-38. doi:10.1111/jpi.12093
26. Kocadağlı T, Yılmaz C, Gökmen V. Determination of melatonin and its isomer in foods by liquid chromatography tandem mass spectrometry. Food Chem 2014;153:151-156. doi:10.1016/j.foodchem.2013.12.036
27. Tan DX, Zanghi BM, Manchester LC, Reiter RJ. Melatonin identified in meats and other food stuffs: potentially nutritional impact. J Pineal Res 2014;57(2):213-218. doi:10.1111/jpi.12152
28. Arnao MB. Phytomelatonin: discovery, content, and role in plants. Adv Botany 2014;2014:815769. doi:10.1155/2014/815769
29. Karunanithi D, Radhakrishna A, Sivaraman KP, Biju VM. Quantitative determination of melatonin in milk by LC-MS/MS. J Food Sci Technol 2014;51(4):805-812. doi:10.1007/s13197-013-1221-6
30. Setyaningsih W, Saputro IE, Barbero GF, et al. Determination of melatonin in rice (Oryza sativa) grains by pressurized liquid extraction. J Agric Food Chem 2015;63(4):1107-1115. doi:10.1021/jf505106m
31. Arnao MB, Hernández-Ruiz J. The potential of phytomelatonin as a nutraceutical. Molecules 2018;23(1):238. doi:10.3390/molecules23010238.
32. Zhang H, Liu X, Chen T, et al. Melatonin in apples and juice: inhibition of browning and microorganism growth in apple juice. Molecules 2018;23(3):521. doi:10.3390/molecules23030521
33. Tijero V, Muñoz P, Munné-Bosch S. Melatonin as an inhibitor of sweet cherries ripening in orchard trees. Plant Physiol Biochem 2019;140:88-95. doi:10.1016/j.plaphy.2019.05.007

34. Salehi B, Sharopov F, Fokou PVT, et al. Melatonin in medicinal and food plants: occurrence, bioavailability, and health potential for humans. Cells 2019;8(7):681. doi:10.3390/cells8070681
35. Gomes Domingos AL, Hermsdorff HHM, Bressan J. Melatonin intake and potential chronobiological effects on human health. Crit Rev Food Sci Nutr 2019;59(1):133-140. doi:10.1080/10408398.2017.1360837
36. Paroni R., Dei Cas M, Rizzo J, et al. Bioactive phytochemicals of tree nuts. Determination of the melatonin and sphingolipid content in almonds and pistachios. J Food Compost Anal 2019;82:103227. doi:10.1016/j.jfca.2019.05.010
37. Sangsopha J, Johns NP, Johns J, Moongngarm A. Dietary sources of melatonin and benefits from production of high melatonin pasteurized milk. J Food Sci Technol 2020;57(6):2026-2037. doi:10.1007/s13197-020-04236-5
38. Wang SY, Shi XC, Wang R, et al. Melatonin in fruit production and postharvest preservation: A review. Food Chem 2020;320:126642. doi:10.1016/j.foodchem.2020.126642
39. Wu X, Ren J, Huang X, et al. Melatonin: biosynthesis, content, and function in horticultural plants and potential application. Sci Hortic 2021;288:110392. doi:10.1016/j.scienta.2021.110392
40. Mannino G, Pernici C, Serio G, et al. Melatonin and phytomelatonin: chemistry, biosynthesis, metabolism, distribution and bioactivity in plants and animals-An overview. Int J Mol Sci 2021;22(18):9996. doi:10.3390/ijms22189996.
41. Cruz-Chamorro I, Santos-Sánchez G, Álvarez-Sánchez N, et al. Alcoholic fermentation with Pichia kluyveri could improve the melatonin bioavailability of orange juice. J Funct Foods 2022;99:105325. doi:10.1016/j.jff.2022.105325
42. Verde A, Míguez J. M., Leao-Martins JM, et al. Melatonin content in walnuts and other commercial nuts. Influence of cultivar, ripening and processing (roasting). J Food Compost Anal 2022;105:104180. doi:10.1016/j.jfca.2021.104180
